# Supplementary material for: Orchestrating performance of healthcare networks subjected to the compound events of natural disasters and pandemic
Source: Nat Commun. 2021 Feb 26;12:1338. doi: 10.1038/s41467-021-21581-x (PMC7910591; doi:10.1038/s41467-021-21581-x)
Supplement: Supplementary file 1 — Supplementary Information [file 41467_2021_21581_MOESM1_ESM.pdf]

Supplementary Information: Orchestrating Performance of Healthcare Networks Subjected to the Compound Events of Natural Disasters and Pandemic

Emad M. Hassan, Hussam Mahmoud*

Department of Civil and Environmental Engineering, Colorado State University, Fort Collins, CO, USA
Email: Hussam.Mahmoud@colostate.edu
Tel: 970-491-6605

Supplementary Note 1: Healthcare system model

Healthcare service is measured by a physical metric (e.g., number of available beds) and the level of consumer satisfaction. Hospital functionality can be defined by combining the quantity (Q_V) and quality (Q_S) services [1,2]. The quantity of the offered services is usually estimated based on hospital capacity or the number of staffed beds available for patients based on daily rates [3]. Noteworthy, hospitalization service not only depends on the hospital itself but also on the surrounding lifelines on which the hospital depends. For example, a reduction in transportation network capacity will lead to delays in ambulances' response or even an entire halt to their service. According to Jacques et al. [4], for these beds to be available for use, representing the quantity portion of the service, three main components are required: 1) trained personnel such as physicians, nurses, and supporting staff; 2) qualified space, and 3) sufficient supplies. The quality portion of the offered service, on the other hand, is difficult to describe. Previous studies identified several dimensions to represent the quality of the hospitalization service [5,6]. One way to do so is by defining the service as a function of losses to different hospital departments while considering service redistribution among the departments [4]. The patient waiting time could also be used to represent the quality part of the functionality [2,7]. Previous studies highlighted the impact of transportation network damage on the waiting time and healthcare services after earthquakes [8,9].

To provide a full description of the healthcare system's functionality, Q_H, the quantity of the service, Q_V, is combined with the accessibility, S_A, and effectiveness, S_E, of this service as an indication of the service quality, Q_S, as follows:

$$Q_H(t) = Q_V(t)^{\alpha_V} Q_S(t)^{\alpha_S} = Q_V(t)^{\alpha_V}\{S_A(t)^{\alpha_A} S_E(t)^{\alpha_E}\}^{\alpha_S} \quad (1)$$

Where, α_V and α_S are weighting factors for service quantity and quality, respectively, and α_A and α_E are weighting factors for accessibility and effectiveness of the service, respectively.

To compute the capacity of healthcare facilities, the success tree shown in Supplementary Fig. 1 is utilized. Different success tree is used for each bed type - ER beds, inpatient beds, ICU beds without mechanical ventilators and ICU beds with mechanical ventilator in the investigated facility. The availability condition of each sub-component (basic event) at time t is calculated to estimate the probabilities of different categories of staffed beds availability, $P_{B,em}$, $P_{B,in}$, $P_{B,icu}$, and $P_{B,v}$, which are used to determine the total available number of staffed beds at each healthcare facility. The mean value of different staffed beds availability represents the quantity functionality, Q_V, of this healthcare, which is calculated based on the bed type as:

$$\boldsymbol{E[Q_V(t)]} = \left(\frac{1}{N_{em}}\sum_{n=1}^{N_{em}} P_{B,em}^{n}\right)^{\alpha_{em}} + \left(\frac{1}{N_{in}}\sum_{n=1}^{N_{in}} P_{B,in}^{n}\right)^{\alpha_{in}} + \left(\frac{1}{N_{icu}}\sum_{n=1}^{N_{icu}} P_{B,icu}^{n}\right)^{\alpha_{icu}} + \left(\frac{1}{N_{v}}\sum_{n=1}^{N_{v}} P_{B,v}^{n}\right)^{\alpha_{v}} \quad (2)$$

Where, N_{em}, N_{in}, N_{icu}, and N_v are the numbers of the four bed types - ER beds, inpatient beds, ICU beds without mechanical ventilators and ICU beds with mechanical ventilator in the investigated facility, respectively; and α_{em}, α_{in}, α_{icu}, and α_v are weighting factors for ER beds, inpatient beds, ICU *beds* without mechanical ventilators and ICU beds with mechanical ventilator, respectively.

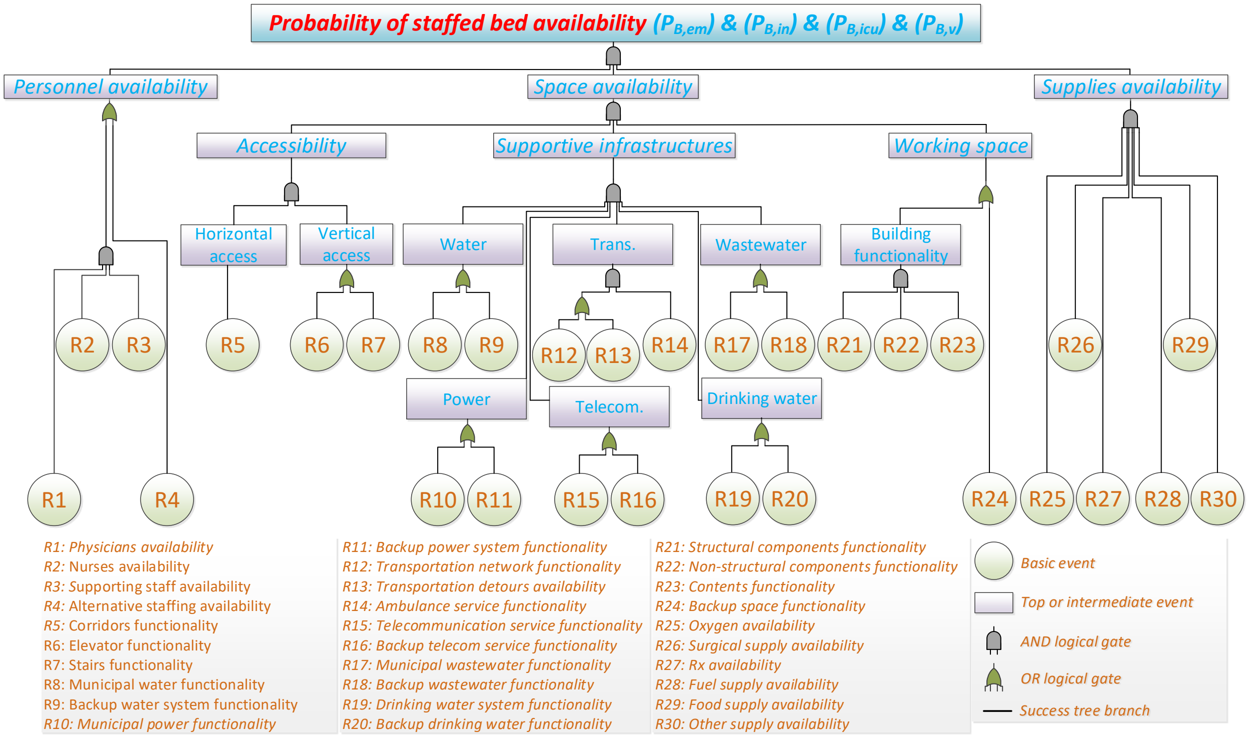

Supplementary Fig. 1. Success tree for determining the availability of staffed beds.

The probability, P, of availability of sub-component, i, in the success tree, P_i, is integrated over the total number of sub-components, n, to define the hospital's probability of receiving patients in terms of the number of available staffed beds, $P_B = \prod_{i=1}^{n} P_i$.

The main differences between these success trees are the calculations of the main events since the availability of staff, space, and supplies between the four types of staffed beds (i.e., units) can be different. The units are modeled to manage their resources independently; however, the interaction among these units is captured by allowing staff and supplies to be transferred from one unit to another and allowing unoccupied beds in one unit to temporarily host patients from other units under certain conditions. This allows, for example, any patient who needs hospital admission and cannot be transferred to other hospitals to temporarily use the ER bed until the inpatient bed becomes available. The same approach applies to space and supplies. In addition, weighting factors are assigned to the success trees' main events to consider the required sub-components for each bed type. Specifically, we remove the non-essential sub-components such as the food and surgical supplies for the ER and increase the weighting factor for the protective supplies, included in the other supplies category, for the facilities treating the epidemic cases.

The quality of the hospitalization service, on the other hand, is difficult to describe and/or quantify. Several dimensions have been identified in previous studies to represent the quality of the

hospitalization service [5,6]. The patient waiting time could also be used to describe the quality part of the functionality [2,7]. In this study, accessibility and effectiveness are utilized to determine the quality of the medical service. Accessibility of the medical services, S_A, is calculated as follows:

$$S_A = [W_t^{max} - W_t^a)]/[W_t^{max} - W_t^b] \geq 0.0 \quad (3)$$

Where, W_t^a and W_t^b are the patient waiting time after and before the disaster, while W_t^{max} is the maximum allowable waiting time. W_t^a is calculated at any time t as follow:

$$W_t^a = W_0 + T_{tvl} + a_t[B_0 - B_t]/B_0 + a_e[N_t - N_0]/N_0 \quad (4)$$

Where, W_0 is the basic waiting time, T_{tvl} is the patient travel time, a_t is the effect of staffed beds reduction, and a_e is the effect of the increase in the total patients' number. W_0 is a function of the patient priority, which is decided upon using the utilized dynamic triage where patients requiring urgent medical services will have less basic waiting time compared with other patients with mild conditions.

To estimate the effectiveness of the offered medical service, S_E, the variation in patient's treatment time is utilized. In which the ratio between the patient treatment time after, T_t^a, and before, T_t^b, the wildfire and epidemic is used, while considering the minimum allowable treatment time, T_t^{min}, as follow:

$$S_E = [T_t^a - T_t^{min})]/[T_t^b - T_t^{min}] \geq 0.0 \quad (5)$$

Patient treatment time, T_t, can be changed after disasters and epidemics due to the higher demand on healthcare facilities and may significantly decrease patient outcomes. T_t is determined at any time t as follow:

$$T_t^a = f(R1^t/N_n^t, PCC^t, SP^t/N_n^t) \quad (6)$$

Where, *R1* is the available physician, N_n is the current hospital demand, *PCC* is the patient case criticality, and *SP* is the available space. We also considered that hospitals will avoid the unnecessary reduction in treatment time to maintain high medical quality and to decrease fatalities related to this treatment time reduction.

Supplementary Note 2: Patient-driven model

Understanding the patient demand on healthcare facility especially during and after natural disasters and pandemics is critical. Different models were previously introduced to investigate hospital choice for patients [10–14], which highlighted the effect of travel time and hospital capacity, among other factors, on patients' choice of the healthcare facility. While many of such models were developed for communities in normal conditions, the model utilized in this study accounts for the disturbance that might occur after natural disasters, including population dislocation and change in hospital capacity. The patient-driven model [15], which is a patient-centric health-seeking model, is used to estimate the patient demand on healthcare facilities. This model calculates the probability $p_{i,n}$ of a patient *i* going to a healthcare facility *n* using different factors that affect the selection of a healthcare facility, as shown in Supplementary Fig. 2.

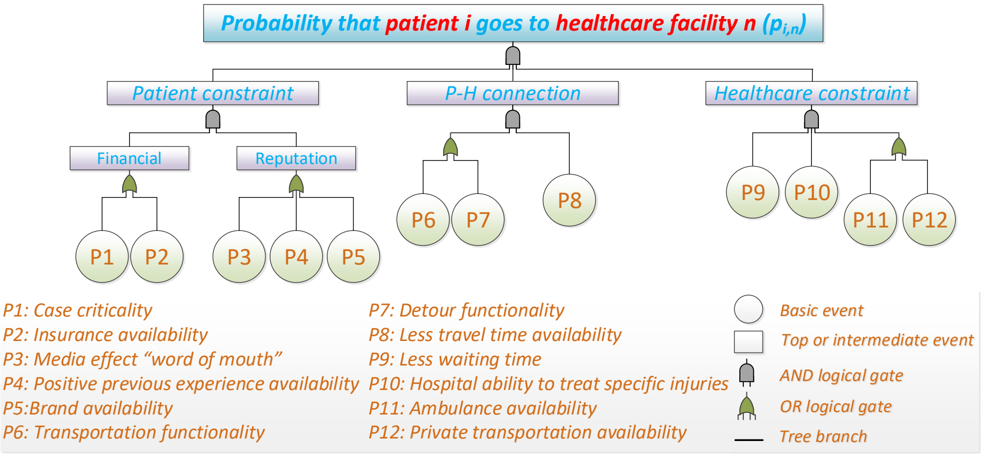

Supplementary Fig. 2. Patient-driven model probability.

The patient selection probability vector $\boldsymbol{P_p}$ is built for all healthcare facilities in the investigated community, N as $\boldsymbol{P_p(t)} = [p_{i,1}\ p_{i,2}\ p_{i,3} \cdots\ p_{i,N}] = p_{i,n}$. The healthcare facility with the highest probability is considered to be the selected hospital by a patient, $\lambda_{i,n}$. Assuming that the community has a total number of patients, N_t, then the expected number of patients, N_n, at a facility, n, can be estimated as the expected total number of patients who will select this hospital as $E[N_n(t)] = \sum_{i=1}^{N_t} \lambda_{i,n}$. The expected demand for healthcare facilities might change further due to the patient transfer process, which is discussed in the next section.

$$\lambda_{i,n} = \begin{cases} 1.0 \iff (Max_{n\epsilon[1:N]} p_{i,n} - p_{i,n} = 0.0) \\ 0.0 \iff (Max_{n\epsilon[1:N]} p_{i,n} - p_{i,n} \neq 0.0) \end{cases} \tag{7}$$

Supplementary Note 3: Healthcare facilities interaction model

The interaction between healthcare facilities is essential, especially during and after natural disasters and pandemics as it allows for redistribution of services, repair resources, medical staff, and patients as needed 16. However, to achieve this level of interaction pre-arranged agreements between healthcare facilities have to exist 17, which is more likely for facilities with the same brand name. The travel distance between the healthcare facilities and the availability of transportation and telecommunication networks can also affect this interaction. The probability, $p_{m,n}$, of patient transfer from a healthcare facility m to facility n is calculated using the probability tree shown in Supplementary Fig. 3.

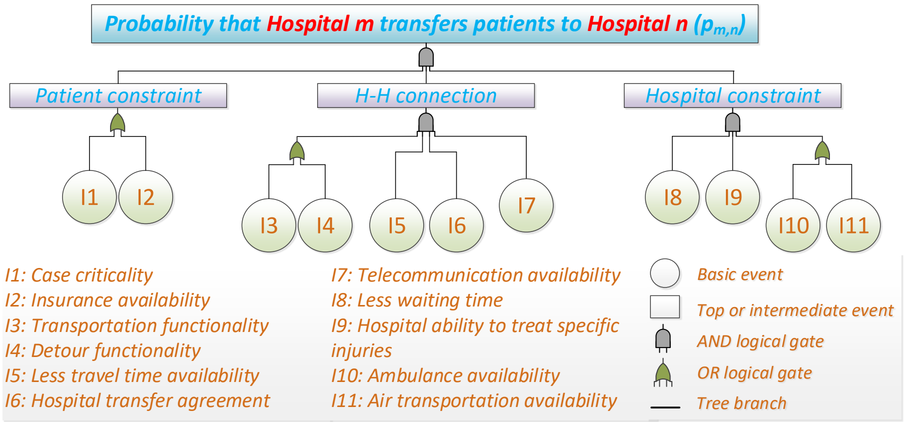

Supplementary Fig. 3. Hospital interaction probability.

The total number of patients transferred, $N_{dist\ m\to n}$, from hospital *m* to hospital *n*, is calculated based on the following equation.

$$N_{dist_{m\to n}}(t) = \left(N_m(t) - \varepsilon_m(t)\right) \frac{p_{m,n}(t)}{\sum_{k=1}^{N}\{p_{m,k}(t)\}} \quad \forall\, n, m \tag{8}$$

Where, N_m is the number of patients at hospital *m*; ε_m is the maximum capacity of the hospital *m* at time t; $p_{m,n}$ is the interaction value between hospital *m* and hospital *n*; and *N* is the number of hospitals that can receive the transferred patient.

Healthcare facilities transfer staff to close the staff shortage gap in other facilities. To estimate the number of additional staff, $M_{s\ m\to n}$, transferring from hospital *m* to hospital *n*, Eq. (9) is used.

$$M_{s_{m\to n}}(t) = \begin{cases} \left(ST_{req_n}(t) - ST_n(t)\right) * \frac{p_{ST_{m,n}}(t)}{\sum_{k=1}^{N}\{p_{ST_{m,k}}(t)\}} \Leftrightarrow ST_n < \min\,(SP_n, SU_n) \\ 0.0 \Leftrightarrow ST_n \geq \min\,(SP_n, SU_n) \end{cases} \quad \forall\, n, m \tag{9}$$

Where, ST_{req_n} is the required number of staff at hospital *n*; ST_n, SP_n, and SU_n are the staff, space, and supplies availability at hospital *n*, respectively; and $p_{ST_{m,n}}$ is the probability of staff transfer from hospital *m* to hospital *n*.

$$p_{ST_{m,n}}(\mathrm{t}) = p\left(E_{ag_{m,n}} \cap E_{tf_{m,n}} \cap E_{ma_{m,n}} \middle| ST_m > \min\,(SP_m, SU_m)\right) \tag{10}$$

$p_{ST_{m,n}}$ is calculated based on the expected values of agreement availability, E_{ag}, between hospital *m* and *n*, the hospital *m* staff accepting a transfer, E_{tf}, and the transferred staff matching the need of hospital *n*, E_{ma}. However, this transfer will occur if and only if hospital *m* has more staff than required.

Healthcare facilities can also transfer supplies, as shown in Eq. (11). The number of supplies, $SU_{add\ m\to n}$, transferred from hospital *m* to hospital *n* can be described as follows:

$$SU_{add_{m\to n}}(t) = \begin{cases} \left(SU_{req_n}(t) - SU_n(t)\right) * \frac{p_{SU_{m,n}}(t)}{\sum_{k=1}^{N}\{p_{SU_{m,k}}(t)\}} \Leftrightarrow SU_n < \min\,(ST_n, SP_n) \\ 0.0 \Leftrightarrow SU_n \geq \min\,(ST_n, SP_n) \end{cases} \quad \forall\, n, m \tag{11}$$

Where, SU_{req} and SU_n are the required and current amount of supplies at hospital *n*, respectively, *and* p_{SU} is the probability of the supplies transfer and is calculated as follow:

$$p_{SU_{m,n}}(\mathrm{t}) = p\left(E_{ag_{m,n}} \cap E_{tr_{m,n}} \cap E_{ma_{m,n}} \middle| SU_m > \min\left(ST_m, SP_m\right)\right) \tag{12}$$

Values of p_{SU} depend on the established agreement, E_{ag}, transportation functionality between hospital *m* and hospital *n*, E_{tr}, and the supplies matching the hospital needs, E_{ma}. However, this transfer will occur if and only if hospital *m* has more supplies than required.

Supplementary Note 4: Results

Change in the patient distribution in Butte County after the Feather River Hospital evacuation is shown in Supplementary Fig. 4.

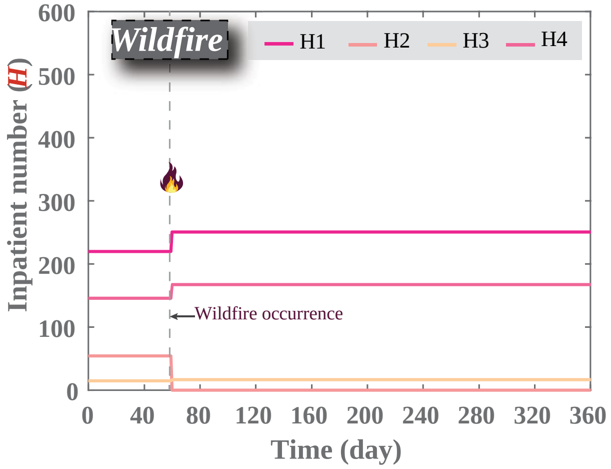

Supplementary Fig. 4. The distribution of inpatient cases on hospitals in Butte County for the case of wildfire.

Curve fitting for the modified SEIR model for active, recovered, and deceased cases for six different regions are shown in Supplementary Fig. 5, where the active cases refer to the cases tested positive for COVID-19 (i.e., confirmed cases).

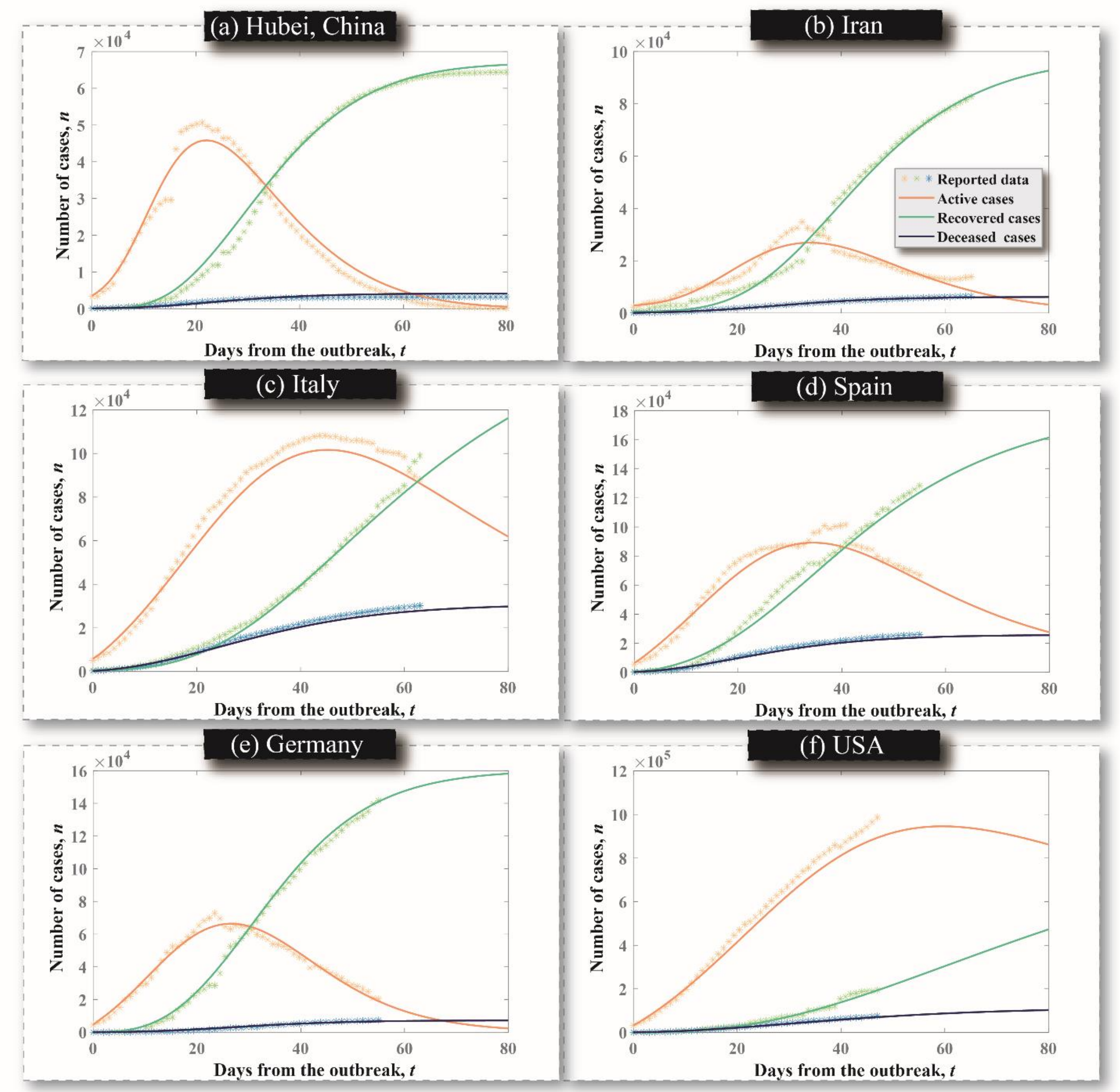

Supplementary Fig. 5. SEIR model parameters fitting to different COVID-19 epidemic in **a**) Hubei, China; **b**) Iran; **c**) Itlay; **d**) Spain; **e**) Germany; and **f**) the USA. The daily reported data for active, recovery and deceased cases in these locations from the initial outbreak to 7th May 2020 are used [18].

Comparing the distributions of the epidemic-related patients, calculated using hospitalization parameters from the Center for Disease Control (CDC) [28] and the European Center for Disease Prevention (ECDC) [31], shows an agreement between the estimated cases that need inpatient, ICU, and mechanical ventilation services, as shown in Supplementary Fig. 6. The figure also displays the envelope for the 2.5 and 97.5 percentiles for each hospitalization service.

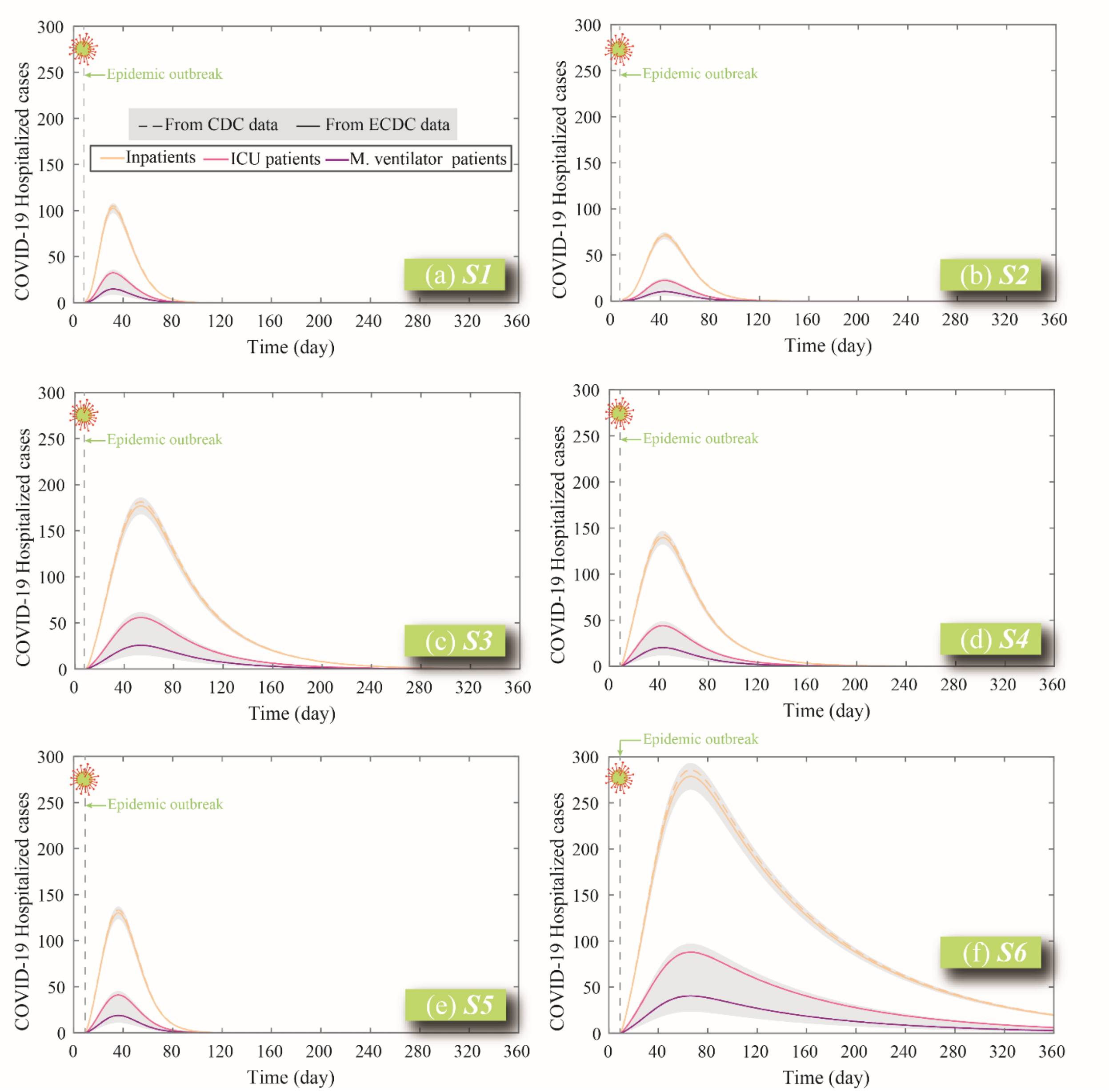

Supplementary Fig. 6. The distribution of COVID-19 related patients classified based on the hospitalization service needed for different disease transmission scenarios including **a**) Hubei, China (S1), **b**) Iran (S2), **c**) Italy (S3), **d**) Spain (S4), **e**) Germany (S5), and **f**) the U.S. (S6). These figures also show the 2.5 and 97.5 percentiles for hospitalization cases calculated using Monte-Carlo simulations with 100,000 trials.

The changes in the inpatients' distribution for the hospitals located in Butte County for the case of no wildfire and different COVID-19 spreads are displayed in Supplementary Fig. 7.

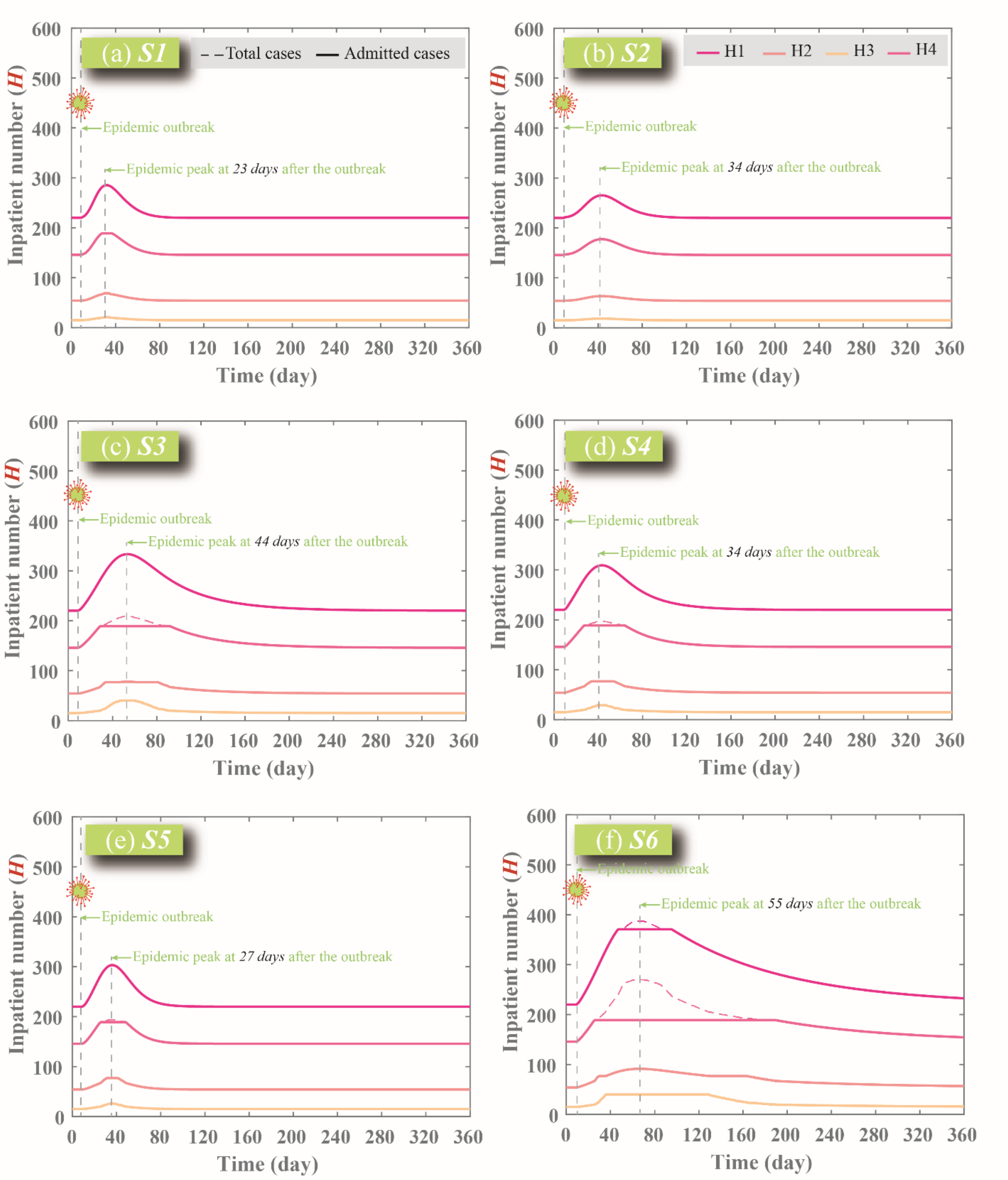

Supplementary Fig. 7. The distribution of inpatient cases for hospitals in Butte County for the case of different disease transmission scenarios including **a**) Hubei, China (S1), **b**) Iran (S2), **c**) Italy (S3), **d**) Spain (S4), **e**) Germany (S5), and **f**) the U.S. (S6).

Supplementary Note 5: Model verification and Input parameters

A comparison between the distribution of the most probable hospital per census tract for Butte County before the occurrence of wildfire and pandemic using the utilized patient-driven model [15] and a model developed by Jia et al. [13] is shown in Supplementary Fig. 8. The Jia et al. [13] model was

developed based on 2,376,743 inpatient discharge records from 22 acute, long-term care hospitals and 199 general medical and surgical hospitals were extracted for Florida in 2011.

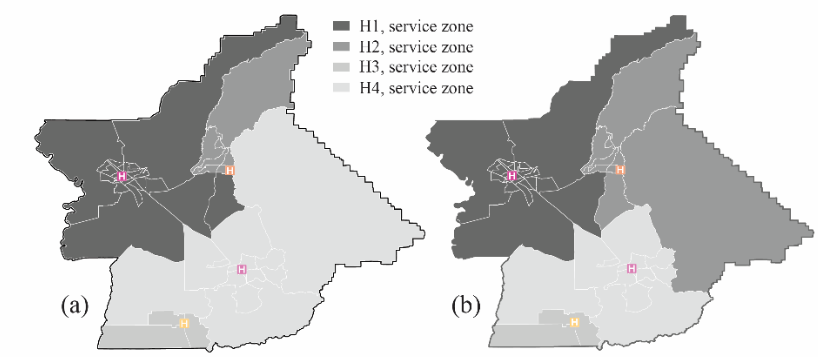

Supplementary Fig. 8**.** The distribution of the most probable hospital per census tract for Butte County a) using the patient-driven model [15] and b) using the gravity model developed by Jia et al. [13].

The Butte County healthcare system model parameters are shown in Supplementary Table 1. The disease transmission model parameters and hospitalization rates for the utilized SEIR disease transmission model are displayed in Supplementary Table 2 and Supplementary Table 3, respectively.

Supplementary Table 1**:** Healthcare system model parameters for Butte County.

**Model parameter**	**Healthcare facility**				**Source**
	**Enloe Medical Center (H1)**	**Feather River Hospital (H2)**	**Orchard Hospital (H3)**	**Oroville Hospital (H4)**	
Facility type	Acute care	Acute care	Acute care	Acute care	OSHPD [19]
Emergency department	Standard	Standard	Stand by	Standard	OSHPD [20]
City	Chico	Paradise	Gridley	Oroville	OSHPD [19]
Number of staffed beds	298	100	45	133	Community benefits reports [21,22]
Ambulance service	Ground & Air	Ground	Ground	Ground	Community benefits report [21,22]
Rating (out of 5)	4.7	4.1	3.85	4.1	Data sets [23–25]
Patient experience	Good	Average	Not available	Fair	Hospital Care Data [25]
Agreement to transfer patients	√	√	√	√	Community benefits reports [21,22]
Average waiting time (min)	49	32	30[a]	60	Data sets [23–25]
Backup systems	√	√	√	√	Community benefits report [21,22]
Emergency department visits/y (2018)	57663	21891	11302	10406	OSHPD [20]

Hospital admission/y (2018)	13256	2584	12066	246	OSHPD [20]
Ratio of total acute care admissions from January to May 2018	0.51	0.13	0.01	0.35	OSHPD [26]
Average length of stay (days)	4.77	4.0[a]	3.5	3.94	American Hospital Directory [27]
Accepted insurance	Private and public	Private and public	Private and public	Private and public	American Hospital Directory [27]

[a] Data not available and assumed based on other data sources

Supplementary Table 2**:** Disease transmission model parameters.

Parameter	Age group	Value	Source
Percentage of infections requiring hospitalization	<19y	1.6	CDC [28]
	20-44y	14.3	
	45-54y	21.2	
	55-64y	20.5	
	65-74y	28.6	
	75-84y	30.5	
	>85y	31.3	
Percentage of infections admitted to ICU	<19y	0.0	CDC [28]
	20-44y	2.0	
	45-54y	5.4	
	55-64y	4.7	
	65-74y	8.1	
	75-84y	10.5	
	>85y	6.3	
Hospitalized patients' median length of stay (days)	All	12	Zhou et al. [29] and Weissman et al. [30]
ICU patients' median length of stay (days)	All	8	Zhou et al. [29] and Weissman et al. [30]
Percentage of ICU stay on mechanical ventilation	All	75	Weissman et al. [30]

Supplementary Table 3**:** Hospitalization rates' parameters, distributions, and ranges.

Parameter	Age group	Distribution	Percentile			Source
			2.5%	50%	97.5%	
Proportion of infections requiring hospitalization	<10y	Normal (μ=0.096, σ=0.011)	0.075	0.096	0.117	ECDC [31]
	10-20y	Normal (μ=0.059, σ=0.007)	0.044	0.059	0.074	
	20-30y	Normal (μ=0.064, σ=0.006)	0.051	0.064	0.077	
	30-40y	Normal (μ=0.088, σ=0.005)	0.077	0.088	0.098	
	40-50y	Normal (μ=0.120, σ=0.006)	0.107	0.120	0.133	
	50-60y	Normal (μ=0.160, σ=0.005)	0.151	0.161	0.171	
	60-70y	Normal (μ=0.300, σ=0.007)	0.288	0.303	0.318	
	70-80y	Normal (μ=0.490, σ=0.009)	0.474	0.491	0.508	
	80-90y	Normal (μ=0.400, σ=0.007)	0.385	0.400	0.415	
	>90y[a]	Normal (μ=0.400, σ=0.007)	0.385	0.400	0.415	
	<10y	Normal (μ=0.029, σ=0.001)	0.027	0.029	0.031	

Proportion of hospitalizations admitted to ICU	10-20y	Normal (μ=0.025, σ=0.001)	0.023	0.025	0.027	ECDC [31]
	20-30y	Normal (μ=0.025, σ=0.001)	0.023	0.025	0.027	
	30-40y	Normal (μ=0.027, σ=0.001)	0.025	0.027	0.030	
	40-50y	Normal (μ=0.031, σ=0.001)	0.029	0.031	0.033	
	50-60y	Normal (μ=0.042, σ=0.001)	0.040	0.042	0.044	
	60-70y	Normal (μ=0.069, σ=0.004)	0.061	0.070	0.077	
	70-80y	Normal (μ=0.101, σ=0.008)	0.086	0.101	0.117	
	80-90y	Normal (μ=0.065, σ=0.007)	0.051	0.065	0.079	
	>90y[a]	Normal (μ=0.065, σ=0.007)	0.051	0.065	0.079	
Proportion of ICU patients requiring ventilation	All	Beta (base= 0.46, location= 5.22, scale= 3.08)	0.299	0.640	0.900	Zhou et al. [29]

[a] Statistical distribution assumed based on other age groups

Supplementary References

1. Cimellaro, G. P., Reinhorn, A. M. & Bruneau, M. Performance-based metamodel for healthcare facilities. 1197–1217 (2011) doi:10.1002/eqe.
2. Hassan, E. M. & Mahmoud, H. Full functionality and recovery assessment framework for a hospital subjected to a scenario earthquake event. *Eng. Struct.* **188**, 165–177 (2019).
3. Denver Health. *National Hospital Available Beds for Emergencies and Disasters (HAvBED) System: Final Report. AHRQ Publication No. 05-0103*. (2005).
4. Jacques, C. C. *et al.* Resilience of the Canterbury hospital system to the 2011 Christchurch earthquake. *Earthq. Spectra* **30**, 533–554 (2014).
5. Kalaja, R., Myshketa, R. & Scalera, F. Service quality assessment in health care sector: The case of Durres public hospital. *Procedia - Soc. Behav. Sci.* **235**, 557–565 (2016).
6. Maxwell J. R. Perspectives in NHS Management: Quality assessment in health. *Br. Med. J.* **288**, 1470–1472 (1984).
7. Mccarthy, K., Mcgee, H. M. & Boyle, C. A. O. Outpatient clinic waiting times and non-attendance as indicators of quality. *Psychol. Health Med.* **5**, 287–293 (2010).
8. Dong, Y. & Frangopol, D. Probabilistic assessment of an interdependent healthcare–bridge network system under seismic hazard. *J. Struct. Infrastruct. Eng.* **13**, 160–170 (2017).
9. Lupoi, A., Cavalieri, F. & Franchin, P. Seismic resilience of regional health-care systems. in *11th International Conference on Structural Safety & Reliability* 4221–4228 (2013). doi:10.1201/b16387-611.
10. Sivey, P. The effect of waiting time and distance on hospital choice for English cataract patients. *Health Econ.* **21**, 444–456 (2012).
11. Raval, D. & Rosenbaum, T. Why is distance important for hospital choice? Separating home bias from transport costs. *Work. Pap.* 1–52 (2019).
12. Adams, E. K., Houchens, R., Wright, G. E. & Robbins, J. Predicting hospital choice for rural Medicare beneficiaries: the role of severity of illness. *Health Serv. Res.* **26**, 583–612 (1991).
13. Jia, P., Wang, F. & Xierali, I. M. Differential effects of distance decay on hospital inpatient visits among subpopulations in Florida, USA. *Environ. Monit. Assess.* **191**, (2019).
14. Smith, H., Currie, C., Chaiwuttisak, P. & Kyprianou, A. Patient choice modelling: how do patients choose their hospitals? *Health Care Manag. Sci.* **21**, 259–268 (2018).
15. Hassan, E. M. & Mahmoud, H. An integrated socio-technical approach for post-earthquake recovery of interdependent healthcare system. *Reliab. Eng. Syst. Saf.* **201**, (2020).
16. Mcdaniels, T., Chang, S., Cole, D., Mikawoz, J. & Longstaff, H. Fostering resilience to extreme events within infrastructure systems: Characterizing decision contexts for mitigation and adaptation. *Glob. Environ. Chang.* **18**, 310–318 (2008).
17. Paterson, J., Berry, P., Ebi, K. & Varangu, L. Health care facilities resilient to climate change impacts. *Int. J. Environ. Res. Public Health* **11**, 13097–13116 (2014).
18. Dong, E., Du, H. & Gardner, L. An interactive web-based dashboard to track COVID-19 in real time. *Lancet Infect. Dis.* **3099**, 19–20 (2020).
19. Office of Statewide Health Planning and Development. OSHPD Healthcare Facilities. https://oshpd-chhsagency.opendata.arcgis.com/datasets/3f0036c60fa04c9ea50251ba92913db8_0?geometry=-150.594%2C31.057%2C-88.060%2C43.264 (2018).

20. Office of Statewide Health Planning and Development. *Hospital Emergency Department – Encounters by Facility*. https://data.chhs.ca.gov/dataset/5b5ec987-692c-4b63-a6cb-ef97db518ee7/resource/eb592e95-d6bd-4ec7-9493-840978188c88/download/encounters-by-facility_2012-18_20190628.csv (2018).
21. Enloe Medical Center. *Community benefits report*. (2018).
22. Orchard Hospital. *Community health needs assessment.* (2016).
23. HospitalStats.org. Hospital Stats. *Butte County Hospitals* https://www.hospitalstats.org/hospital-ratings/ (2020).
24. ER Inspector. California ER Wait Times & Violations. https://projects.propublica.org/emergency/state/CA (2020).
25. Hospital Care Data. https://hospitalcaredata.com/ (2020).
26. Office of Statewide Health Planning and Development. OSHPD report center: Facility summary reports. https://report.oshpd.ca.gov/ (2018).
27. American Hospital Directory. Individual Hospital Statistics for California. https://www.ahd.com/states/hospital_CA.html (2020).
28. CDC COVID-19 Response Team. *Severe Outcomes Among Patients with Coronavirus Disease 2019 (COVID-19) - United States, February 12-March 16, 2020*. *MMWR. Morbidity and mortality weekly report* vol. 69 (2020).
29. Zhou, F. *et al.* Clinical course and risk factors for mortality of adult inpatients with COVID-19 in Wuhan, China: a retrospective cohort study. *Lancet* **395**, 1054–1062 (2020).
30. Weissman, G. E. *et al.* Locally Informed Simulation to Predict Hospital Capacity Needs During the COVID-19 Pandemic. *Ann. Intern. Med.* (2020) doi:10.7326/m20-1260.
31. European Centre for Disease Prevention and Control. *Rapid risk assessment: Increased transmission of COVID-19 in the EU/EEA and the UK-twelfth update*. https://www.ecdc.europa.eu/en/novel- (2020).
